# A value accumulation account of unhealthy food choices: testing the influence of outcome salience under varying time constraints

**DOI:** 10.1186/s41235-022-00459-6

**Published:** 2023-01-12

**Authors:** Massimo Köster, Eike K. Buabang, Tina Ivančir, Agnes Moors

**Affiliations:** 1 Research Group of Quantitative Psychology and Individual Differences, KU Leuven, Leuven, Belgium; 2 Center for Social and Cultural Psychology, KU Leuven, Leuven, Belgium; 3 Trinity College Institute of Neuroscience, Trinity College Dublin, Dublin, Ireland

**Keywords:** Food choice, Unhealthy eating, Goal-directed, Value accumulation, Dual-process models

## Abstract

People often engage in unhealthy eating despite having an explicit goal to follow a healthy diet, especially under certain conditions such as a lack of time. A promising explanation from the value accumulation account is that food choices are based on the sequential consideration of the values of multiple outcomes, such as health and taste outcomes. Unhealthy choices may result if taste is considered before health. We examined whether making a health outcome more salient could alter this order, thereby leading to more healthy choices even under time pressure. Two studies examined the time-dependent effect of outcome values and salience on food choices. Participants first completed priming trials on which they rated food items on healthiness (health condition), tastiness (taste condition), or both healthiness and tastiness (control condition). They then completed blocks of binary choice trials between healthy and tasty items. The available response time was manipulated continuously in Study 1 (*N* = 161) and categorically in Study 2 (*N* = 318). As predicted, results showed that the values of health and taste outcomes influenced choices and that priming led to more choices in line with the primed outcomes even when time was scarce. We did not obtain support for the prediction that the priming effect is time-dependent in the sense that primed outcomes are considered before non-primed outcomes. Together, these findings suggest that increasing the value and salience of a health outcome may be effective ways to increase healthy choices, even under poor conditions such as time pressure.

**Supplementary Information:**

The online version contains supplementary material available at 10.1186/s41235-022-00459-6.

## Significance statement

Obesity is a serious issue at the societal and individual level, resulting in economic costs and adverse health consequences. A key step in addressing this issue is the adoption of a healthy diet. While many people wish to adopt a healthy diet, they often fail to do so. This seems to be especially the case when people are confronted with a lack of opportunity, capacity, or motivation, such as when pressed for time or under stress. In order to find effective strategies to promote healthy eating, it is important to study the underlying mechanisms of food choice under varying conditions. In the current research, we examined how food choices under varying time constraints are influenced by the values and salience of peoples’ health and taste goals. We found that people generally made food choices in line with their health and taste goals. Moreover, temporarily increasing the salience of the health goal promoted healthy choices and, importantly, this was also the case when people were pressed for time. An important implication of these findings is that healthy eating may be promoted by making health more salient in everyday contexts even when people are pressed for time.

## Introduction

According to the World Health Organization, obesity has nearly tripled since 1975. In 2016, 39% of adults worldwide were overweight and 13% were obese (World Health Organization, 2021). Obesity is a serious issue at the global and individual level because it increases the risk of diabetes, cardiovascular disease, musculoskeletal disorders, and some types of cancer (Segula, 2014). A crucial factor to decrease overweight prevalence and its associated risks is a healthy diet (Micha et al., 2017). Many individuals are trying to improve their eating patterns, and there seems to be a growing awareness about the importance of a healthy diet (Anna, 2001; Food Insight, 2018). Yet people often fail to adopt a healthy diet especially when confronted with a lack of opportunity, capacity, or motivation. For instance, it has been frequently reported that time constraints (Andajani-Sutjahjo et al., 2004; Escoto et al., 2012; Greaney et al., 2009) and stress (e.g., O’Connor et al., 2008) can lead to unhealthy eating. In order to effectively combat unhealthy eating, it is crucial to understand the underlying mechanisms. In the current research, we aim to shed light on the mismatch between individuals’ explicit health goals and their unhealthy food choices and offer an explanation for why unhealthy eating may be more frequent when time is limited.

## Traditional dual-process account of unhealthy food choices

To explain unhealthy eating, researchers often turn to dual-process models, which distinguish between goal-directed and stimulus-driven processes (Hofmann et al., 2009; Wood & Rünger, 2016). In a goal-directed process, available behavior options are evaluated in terms of their expected utilities, that is, how likely they are to reach a person’s valued outcomes. Here, the behavior option with the highest expected utility activates the tendency to engage in the corresponding behavior and ultimately the behavior itself. In a stimulus-driven process, on the other hand, behavior is determined via the activation of a preexisting association between the representation of a stimulus and the representation of a response or action tendency (short, an S–R association). The traditional dual-process account explains unhealthy eating despite the presence of explicit health goals as the result of a stimulus-driven process. This is based on the idea that stimulus-driven processes do not take the impact of behavior options on valued outcomes into account like goal-directed processes do. More precisely, unhealthy eating is driven by a stimulus-driven process in which tasty but unhealthy food stimuli activate an association between this type of food stimuli and the urge to consume them (Hartogsveld et al., 2022; Pierce-Messick & Corbit, 2021).

To explain why unhealthy eating occurs more frequently when time is limited, the traditional dual-process account relies on the corollary assumption that goal-directed and stimulus-driven processes occur under different operating conditions. Stimulus-driven processes are assumed to be automatic, which means that they can operate when conditions are poor such as when there is a lack of time, attention, and/or motivation (Moors, 2016). This means that stimulus-driven processes operate fast, when the individual is distracted, and/or when the stakes are low. Goal-directed processes are assumed to be non-automatic, which means that they require ample conditions to operate, such as ample time, attention, and motivation. The implication is that tasty but unhealthy food triggers a fast, initial urge to consume it and that people can only act in line with their health goal if they recruit a goal-directed process that overrides this stimulus-driven process. However, since the goal-directed process requires ample operating conditions, attempts to override the urge to eat unhealthy food will only occur when these conditions are indeed ample. When these conditions are poor, such as when time is limited or capacity is reduced because of stress, people have no choice but to rely on a stimulus-driven process, which is more likely to push them into unhealthy eating (e.g., Pool et al., 2015; Schwabe & Wolf, 2009; Smeets et al., 2019).

## Value accumulation of unhealthy food choices

Recently, it has been proposed that goal-directed processes can operate fairly automatically as well, suggesting that most behavior is goal-directed even under poor operating conditions (Moors et al., 2017). However, if most behavior is goal-directed, this begs the question of why people engage in behavior that is not in line with their explicit goals and why this is especially likely to happen under poor operating conditions. A potential explanation comes from sequential accumulation models (see Berkman et al., 2017), such as the decision-field theory (Busemeyer & Townsend, 1993), the leaky competing-accumulator model (Usher & McClelland, 2001), and drift–diffusion models (Ratcliff & McKoon, 2008). These models explain behavior as the result of a dynamic goal-directed process in which behavior options are evaluated based on a dynamic integration of evidence over time. According to Berkman et al. (2017), the integration of evidence is based on a sequential consideration of multiple outcomes^1^ over time. If outcomes are indeed considered in a stepwise fashion, this can explain why people’s decisions under time constraints are not always in line with the overall expected utilities of the behavior options that are compared. The mere order in which outcomes are considered can tip the balance toward one decision if the outcome that is considered first favors one option and value accumulation is stopped early due to time pressure. To illustrate, a person who has to choose between two food items may first evaluate both options in terms of a taste outcome before evaluating them both in terms of a health outcome. If the person makes a decision before the health outcome is considered, unhealthy food choices are especially likely to occur.

Several proponents of accumulation models indeed seem to assume that taste is considered before health and that this is the reason why unhealthy food choices are especially likely to occur under poor operating conditions. The assumption that taste is considered first is often grounded in the idea that taste is a more concrete outcome than health combined with the idea that concrete outcomes are considered faster than abstract outcomes (e.g., Sullivan & Huettel, 2021; Sullivan et al., 2015). Based on this explanation in terms of concreteness, the order of consideration should remain fixed: Taste outcomes should always be considered first and health outcomes should always be considered later. An alternative explanation, proposed by Berkman et al. (2017), is that the order in which outcomes are considered depends on their salience. If taste is typically considered before health, this is because it is typically more salient than health. Based on this explanation in terms of salience, the typical order of taste being considered before health is not fixed but could in principle be reversed if the salience of the health outcome would be increased to exceed that of the taste outcome. Thus, increasing the salience of the health outcome should increase the likelihood that this outcome is considered first (Maier et al., 2020). This should in turn increase the likelihood that the healthy item is selected, especially when operating conditions are poor such as when time is scarce.

The aim of the current research is to examine three key assumptions of the value accumulation account of unhealthy food choices. A first key assumption is that available behavior options are evaluated and chosen based on the consideration of one or multiple outcomes. From this assumption, we can derive the prediction that the values people ascribe to outcomes will influence their choices. A second key assumption is that the salience of an outcome influences the likelihood that it is considered. From this assumption, we can derive the prediction that increasing the salience of an outcome will increase the likelihood that choices are made in line with this outcome. A third key assumption is that the salience of different outcomes affects the order in which these outcomes are considered. In particular, more salient outcomes are more likely to be considered before less salient outcomes. From this assumption, we can derive the prediction that the influence of the salience of an outcome on the likelihood that choices are made in line with this outcome will be stronger when decision time is short than when it is long. This is because a short decision time should increase the likelihood that the outcome for which the salience is increased and therefore considered first is also the only outcome that is considered before a decision is made. A longer decision time, on the other hand, should increase the likelihood that other outcomes are considered in addition to the first outcome.

Existing research already provides substantial evidence for the first two predictions in the food choice domain. Indeed, it has been shown repeatedly that food choices or preferences are value-based (e.g., Krajbich et al., 2015) and influenced by outcome salience (e.g., Hare et al., 2011; Hege et al., 2018). To illustrate, in a study by Hare et al. (2011), outcome salience was manipulated by asking participants to consider either healthiness, tastiness, or to make decisions naturally. To measure food preferences, participants were presented with single food items and had to rate how much they wanted to eat them. Results showed that participants had a stronger preference for healthy items when asked to consider healthiness compared to when they were asked to make decisions naturally. Evidence for the third prediction—that increasing the salience of the health outcome increases the likelihood of being considered first—is mixed. Preliminary support comes from a re-analysis of the Hare et al. (2011) study by Maier et al. (2020) using drift–diffusion modeling. This re-analysis revealed that the effect of the priming of the health outcome on the preference for healthy items was especially pronounced for decisions with fast response times. This suggests that the order in which health and taste are considered depends on their salience. Another recent study by Sullivan and Huettel (2021), by contrast, did not find support for this prediction. In their study, outcome salience was manipulated by asking participants to read about the importance of either health or taste, and food preferences were measured with binary food choices. Results indicated that taste was considered before health and that priming health could not alter this. This suggests a fixed order of consideration in which taste is considered before health.

It is important to note that previous studies have relied on the observation of spontaneous response times. In the study by Hare et al. (2011), participants were allowed to respond at any time within a relatively broad time window (3 s), and in the study by Sullivan and Huettel (2021), participants could choose at their own pace. An advantage of measuring spontaneous response time differences is that they are suitable for fitting drift–diffusion models. A potential disadvantage, however, is that spontaneous response times may not merely reflect the factor time but may be confounded with other factors such as choice difficulty. Choice difficulty has been shown to increase decision times in all kinds of binary choices (Ratcliff & Rouder, 1998; Rolls et al., 2010) and in binary choices between food items in particular (Milosavljevic et al., 2010; Sullivan & Huettel, 2021; Sullivan et al., 2015). In the current research, we wanted to study the influence of time in a more controlled manner. To achieve this, we manipulated available response times instead of comparing spontaneously chosen short and long response times. In this way, the current research attempted to replicate previous findings that food choices depend on the values and salience of health and taste outcomes and to extend previous work that examined the order in which these two outcomes are considered in food choices.

## Current study

We conducted two studies in which participants completed binary choice trials in which they had to choose between a healthy and a tasty food item. In both studies, the salience of health versus taste outcomes was manipulated between participants with the help of priming trials that preceded the choice trials. On priming trials, participants were presented with single food items, which they had to rate either on healthiness (health condition), tastiness (taste condition), or both healthiness and tastiness (control condition). During the choice trials, the available response time was manipulated. This was done using two different paradigms. In Study 1, participants had to make a choice when they heard an auditory cue. The available response time was manipulated in a continuous way by varying the onset of the image relative to the auditory cue so that participants had between 400 and 2000 ms to respond. In Study 2, the available response time was manipulated categorically with a visual response cue, giving them either a short response time requiring a response within 1000 ms or a long response time requiring a response between 2000 and 3000 ms. The continuous manipulation of the available response time in Study 1 allowed us to study its effect across a range of different available response times. The categorical manipulation of this variable in Study 2 allowed us to more easily contrast short vs. long available response times and to test for differences with more statistical power than in Study 1.

In both studies, participants completed rating trials at the end of the study. On these trials, participants saw the same pairs of food items they had seen during choice trials and had to indicate which item they considered to be healthier and which they considered to be tastier. The rating trials were added to verify whether the pairs of food items presented in the choice trials indeed constituted a conflict between health and taste for each participant. Choice trials were only included in subsequent analyses if the results of the rating trials confirmed that one item of the pair presented during these choice trials was rated as healthier and the other as tastier. In both studies, we also measured hunger as well as the values that participants ascribed to health and taste outcomes.

In line with the general predictions of the value accumulation account, we formulated three concrete hypotheses for our studies. The first hypothesis was that participants would make food choices in line with the values they ascribed to health and taste outcomes. In particular, we predicted that participants with higher values for health outcomes would make more healthy choices (Hypothesis 1a) and that those with higher values for taste outcomes would make more tasty choices (Hypothesis 1b). The second hypothesis was that increasing the salience of a health/taste outcome via priming would increase the likelihood that choices would be made in line with this outcome. In particular, we predicted more healthy choices in the health prime condition relative to the control prime condition (Hypothesis 2a) and more tasty choices in the taste prime condition relative to the control prime condition (Hypothesis 2b). The third hypothesis was that choices in line with an outcome that was made more salient via priming would be more likely for trials with short than long response times. In particular, we predicted that the increase of healthy choices in the health prime condition relative to the control prime condition would be larger for short than for long response times (Hypothesis 3a). We also predicted that the increase in tasty choices in the taste prime condition relative to the control prime condition would be larger for short than for long response times (Hypothesis 3b).

## Study 1

### Method

#### Transparency and openness

The method, materials, planned sample size, inclusion criteria, hypotheses, and analysis plan were pre-registered on the Open Science Framework prior to data collection. This preregistration alongside all data and the analysis code are available at https://osf.io/uh78j (Koester et al. 2021). The computerized task was programmed in PsychoPy (Peirce et al., 2019).

#### Participants

The preregistered target sample size for this study was 210 participants. We recruited 212 participants, and after applying the inclusion criteria (see below), the final sample consisted of 161 participants (143 females, Age: *M* = 19.90, *SD* = 3.37) with 52, 58, and 51 participants in the health, taste, and control condition, respectively. A continuation of the data collection to reach the desired target sample size was at this point not possible due to the COVID-19 pandemic. The participants were recruited through the Experiment Management System of the Faculty of Psychology and Educational Sciences at KU Leuven and were either paid 4€ or rewarded with partial course credits. All participants provided informed consent, and the study was approved by the Social and Societal Ethics Committee at KU Leuven (G-2019 10 1766).

#### Inclusion criteria

In line with other studies (e.g., Maier et al., 2015), we recruited participants who affirmed valuing a healthy diet while enjoying unhealthy food items from time to time. In addition, we only recruited participants who did not follow a caloric or vegan diet, and who did not have any food allergies or lactose intolerance. Participants were instructed to refrain from eating within 2 h prior to the study. Further, to ensure sufficient data points per participant, participants were only included in the analyses if they had at least 20 valid choice trials (the average number of valid trials per participant was 44.8). To qualify as a valid choice trial, the healthy item had to be rated as healthier and the tasty item as tastier at the end of the study. This criterion ensured that each choice trial constituted a conflict between a healthy and a tasty item.

#### Materials

Based on a small pilot study, we created a set of 60 pictures that were on average rated as healthy but only moderately tasty as well as 60 pictures that were on average rated as tasty but not healthy. The pictures presented colored food items on a white background, and they were all scaled to the same size (175 × 175 pixels). All food items were vegetarian and directly consumable (e.g., a cooked and sliced beetroot instead of a raw beetroot). The reason to include only vegetarian items was to be able to include vegetarian participants.

#### Procedure

This study was conducted in the laboratory using identical computer setups in individual cubicles. At the start of the study, participants had to indicate how many hours ago they ate their last meal as well as their current hunger level on continuous scales ranging from 0 to 10. The rest of the study was divided into three parts: a part with priming and choice trials, a part with rating trials, and a part with post-experimental questions. Participants were informed that they would receive a randomly selected food item that they had chosen during the task. In this way, the binary choices were not merely hypothetical but incentive compatible to some extent. Independent of the choices they made during the experiment, participants were offered a choice between a real tangerine and a real cookie at the end of the study.

**Fig. 1:**
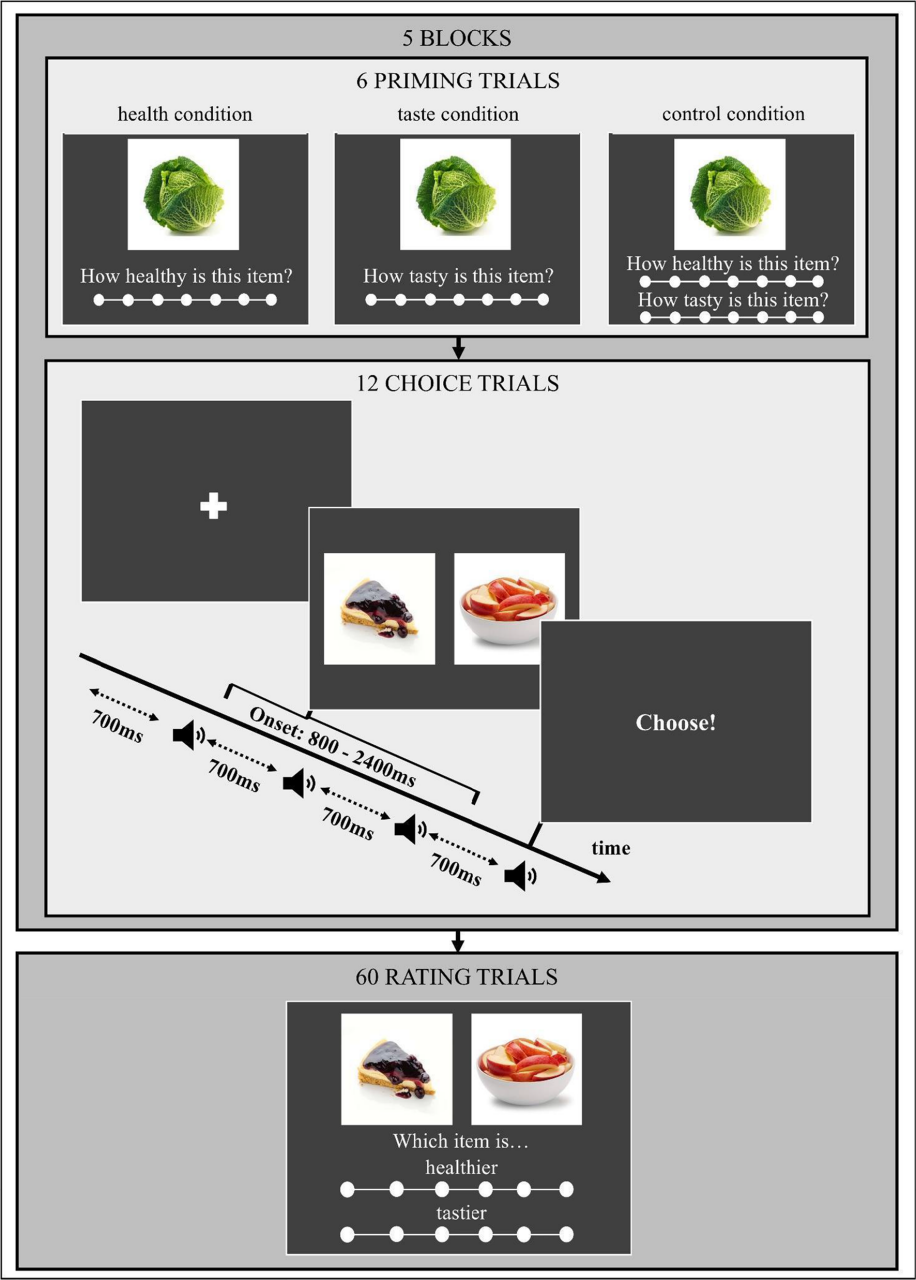
Experimental procedure of Study 1. *Note* The experiment had five blocks, each with six priming trials followed by 12 choice trials. These were followed by one block of 60 rating trials (see text for details)

*Priming and choice trials* In the first part, participants were randomly and unknowingly assigned to a health, taste, or control condition. This part consisted of five blocks, each made up of six priming trials followed by 12 choice trials (see Fig. 1).

On each priming trial, participants were asked to rate a single food item on a 7-point Likert scale. Depending on the priming condition, participants either rated the items on healthiness (health condition), on tastiness (taste condition), or on both healthiness and tastiness (control condition). The six priming trials per block served as a manipulation of outcome salience. Choice trials started with a fixation cross, followed by the presentation of one healthy and one tasty food item arranged horizontally. The location of healthy and tasty items was counterbalanced across trials. The items were randomly selected from the pool of 60 healthy and 60 tasty images. Participants were instructed to choose the left item by pressing the “E”-key and the right item by pressing the “I”-key. The available response time for each choice trial was manipulated with a forced-choice design adapted from Hardwick et al. (2019). On each trial, participants heard a sequence of four equally spaced tones (700 ms). Participants were instructed to choose synchronously with the onset of the fourth tone and thus after 2800 ms. The time for participants to make their choice was manipulated by varying the onset of the food items randomly between 800 and 2400 ms. This means that the available response time varied between 400 and 2000 ms (Fig. 1).

To familiarize participants with the procedure for the choice trials, they completed eight practice trials with non-food items (i.e., flowers) prior to the first block of the actual study. To ensure that participants learned to quickly respond upon hearing the fourth tone, they received a feedback message stating ‘Too slow!’ if responses were given 500 ms or later after the fourth tone.

*Rating trials* The second part of the study consisted of 60 rating trials in which each of the pairs of food items participants had previously seen during the choice trials was presented again and they were asked to indicate on two 6-point Likert scales which item they considered to be healthier and which to be tastier (see Fig. 1). The order in which the pairs were presented and the location of healthy and tasty images on the left or right side of the screen were randomized (and thus not necessarily identical to the way in which they were presented during the choice trials). The rating trials served as a trial-based inclusion criterion to ascertain that each choice trial depicted a choice between a healthy and a tasty item for each participant. Choice trials were only included in the final analysis if a participant indeed rated the preselected healthy item as healthier and the preselected tasty item as tastier.

*Post-experimental questions* During the third part, participants were asked to report their current hunger levels (“How hungry are you at this moment?”), the value they ascribed to a health outcome (“How important is health for you when choosing food items?”), and the value they ascribed to a taste outcome (“How important is taste for you when choosing food items?”) on a continuous scale from 0 (not at all important) to 10 (very important). We chose not to use established scales to measure these outcome values, such as the Adult Eating Behavior Questionnaire (Hunot et al., 2016) or the General Nutrition Knowledge Questionnaire (Kliemann et al., 2016) because these scales do not only measure outcome values but also other, related, aspects such as past experiences and knowledge. Furthermore, the reason for measuring the values of health and taste outcomes at the end and not at the start of the study was to avoid diluting our priming procedure. Finally, participants were asked to report whether they followed a caloric or a vegan diet, had any food allergies, or were lactose-intolerant. Participants who reported any of these dietary restrictions were excluded from the analysis to minimize potential influences of dietary restrictions on food choices.

### Results

#### Descriptive results

##### Health and taste outcome values

To check whether the values of health and/or taste outcomes differed across or between priming conditions, we conducted a mixed-model ANOVA to analyze the self-reported values with priming condition (health, taste, control) as between-subjects factor and outcome value type (health vs. taste) as within-subjects factor.

The main effect of outcome value type was significant, *F*(1, 158) = 122.97, *p* < 0.001, *η*^2^_*p*_ = 0.44, indicating that participants valued a taste outcome (*M* = 8.64, SD = 1.15) more than a health outcome (*M* = 6.82, SD = 1.59). No other effects were significant, all *Fs* < 0.09, suggesting that the values of health and taste outcomes did not differ between the priming conditions.

##### Hunger

To check whether there were differences in hunger between the priming conditions, we conducted a mixed-model ANOVA to analyze the self-reported hunger ratings with priming condition (health, taste, control) as between-subjects factor and time (pre-study vs. post-study) as within-subjects factor.

The main effect of priming condition was significant, *F*(2, 158) = 4.87, *p* = 0.009, *η*^2^_*p*_ = 0.06. Post hoc comparisons indicated that participants in the taste condition (*M* = 7.25, SD = 1.59) reported higher hunger levels than those in the health condition (*M* = 6.28, SD = 2.18), *t*(218) = 3.74, *p* < 0.001, and than those in the control condition (*M* = 6.35, SD = 2.10), *t*(216) = 3.55, *p* < 0.001. The difference in hunger levels between the health and control conditions was not significant, *t*(204) = 0.23, *p* = 0.822. The main effect of time was also significant, *F*(1, 158) = 110.82, *p* < 0.001, *η*^2^_*p*_ = 0.41, indicating that participants were less hungry at the start of the experiment (*M* = 6.20, SD = 1.92) than at the end (*M* = 7.10, SD = 2.00). The interaction between priming condition and time was not significant, *F*(2, 158) = 0.64, *p* = 0.526, suggesting no differences between the conditions over time.

##### Food choices

To analyze the effect of health outcome value, taste outcome value, priming condition, and available response time on food choices, we conducted a multilevel logistic regression. Our regression model had a two-level structure, with trials nested within participants. The outcome variable was the type of choices made by participants on choice trials (healthy = 1, tasty = 0). Available response time was the level 1/within-participant predictor. Health and taste outcome values and priming condition were the level 2/between-participant predictors. Because hunger levels differed between priming conditions, pre-study hunger levels were also added as a predictor. Health outcome value, taste outcome value, and hunger ratings were centered for easier interpretation of the regression results.

Following the steps described by Sommet and Morselli (2017), we compared a constrained intermediate model (CIM) with an augmented intermediate model (AIM). Both models included health outcome value, taste outcome value, priming condition, and pre-study hunger as fixed effects (not varying across participants). The CIM included available response time as a fixed effect, whereas the AIM allowed for participant-specific random slope variance. Here, the results of the AIM are reported, which provided the better fit. The results for the AIM are presented in Table 1. The complete fitting results of all tested models can be found in Additional file 1.

**Table 1:** Results for the augmented intermediate model predicting healthy food choices

Participant-level variables	Category	Augmented intermediate model (AIM)	
		OR (95% CI)	*p*
Priming condition	Control	Reference	
	Health	2.13 (1.43–3.18)	< .001
	Taste	0.58 (0.39–0.88)	.010
Health outcome value		1.26 (1-13–1-40)	< .001
Taste outcome value		0.92 (0.80–1.06)	.253
Hunger		0.96 (0.88–1.05)	.356
Trial-level variables			
Available response time		0.52 (0.43–0.62)	< .001

*OR* odds ratio, *CI* confidence interval, *p*  *p* value

**Fig. 2:**
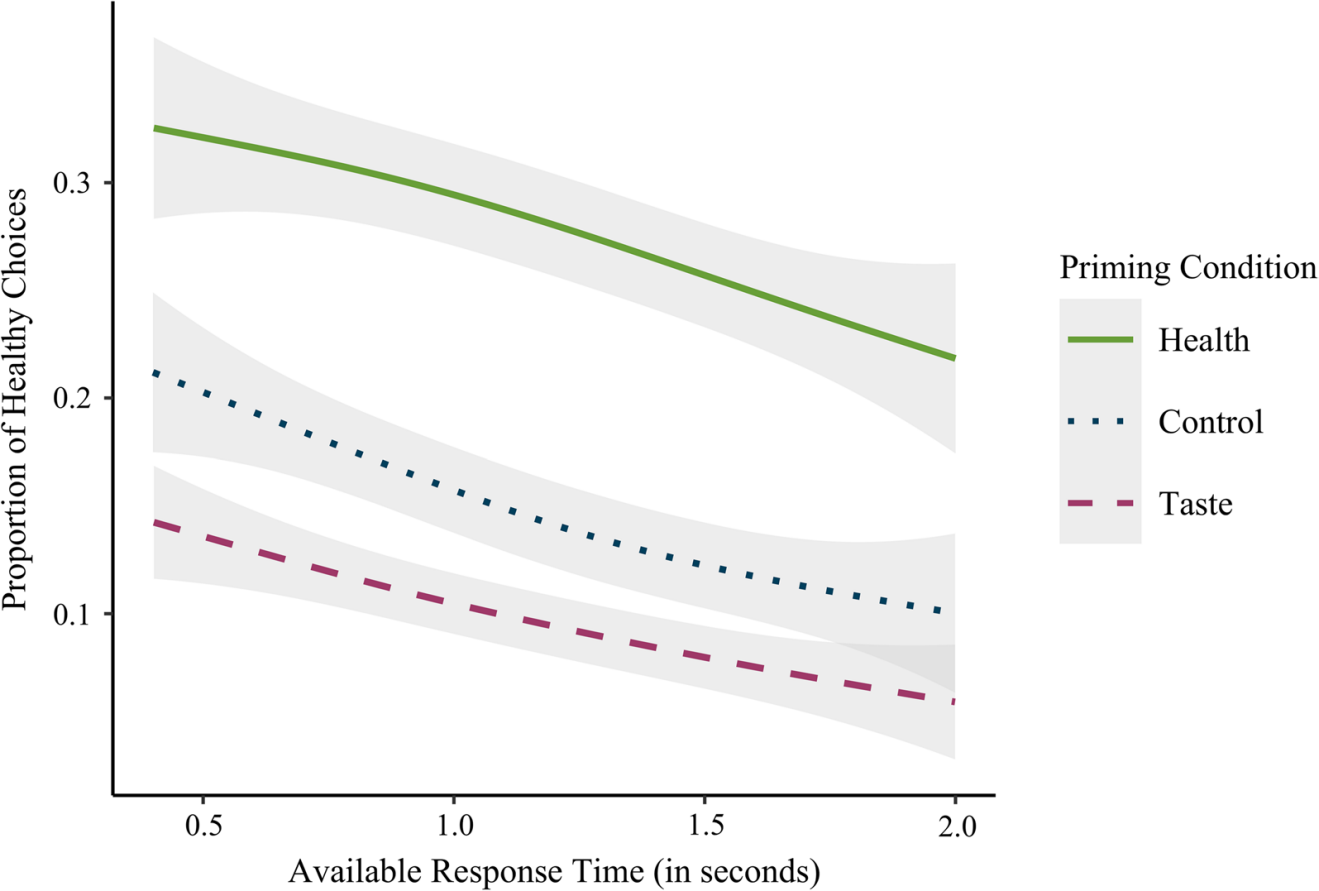
Proportion of healthy choices by available response time and priming condition. *Note* Confidence bands represent 95% confidence intervals

Participants were overall less likely to choose the healthy item than the tasty item (OR 0.31). In line with Hypothesis 1a, the main effect of health outcome value was significant, indicating that participants who reported higher health outcome values were more likely to choose healthy items (OR 1.26). Contrary to Hypothesis 1b, there was no significant main effect of taste outcome value. In line with Hypotheses 2a and 2b, the main effects of health and taste prime were significant (see Fig. 2). In comparison with the control condition, priming with a health outcome increased the odds of selecting healthy items (OR 2.13), whereas priming with a taste outcome decreased the odds of selecting healthy items (OR 0.58). Results also indicated a significant effect of available response time, suggesting that longer available response time decreased the odds of selecting healthy items (OR 0.52; see Fig. 2). To test Hypothesis 3, we created a full model by adding the interaction terms between priming conditions (health vs. control and taste vs. control) and available response time to the AIM. Contrary to this hypothesis, the interactions were not significant. Furthermore, adding the interaction terms to the AIM did not significantly improve the model fit.

### Discussion

We tested three hypotheses derived from the value accumulation account. The first hypothesis, that people make food choices in line with their health and taste values, was partially supported. We found that participants reported higher values for taste than health outcomes and made more tasty than healthy choices overall. This suggests that people make more tasty than healthy choices because they value a taste outcome more than a health outcome. We also found that participants who reported higher health outcome values made more healthy choices. However, contrary to our prediction, we did not find that participants who reported higher taste outcome values made more tasty choices. A potential reason for why food choices were predicted by health but not taste outcome values may be the smaller variance in self-reported taste compared to health outcome values. This may have reduced the explanatory power of taste outcome values.

The second hypothesis, that increasing the salience of a health/taste outcome via priming increases the likelihood that choices are made in line with this outcome, was supported. Indeed, priming participants with only health/taste led to more healthy/tasty choices than priming them with both health and taste.

The third hypothesis, that increasing the salience of a health/taste outcome via priming has a stronger effect on food choices with short relative to long decision times, was not supported. Priming health/taste led to a similar increase in healthy/tasty choices across time. This means that our results do not support the idea that increasing the salience of a health/taste outcome increases the likelihood that the outcome is considered first. A potential limitation of our study was that the number of participants and choice trials per participant may not have been sufficient to detect an interaction effect between levels in a multilevel logistic regression (Schoeneberger, 2016).

One unexpected finding worth mentioning is that, although participants made more tasty choices overall, they made more healthy choices on trials with short relative to long response times. This suggests that the health outcome was more likely to be considered when time was short than when it was long. In the previous research, the opposite pattern is usually observed (e.g., Sullivan & Huettel, 2021). Potential explanations for this finding will be discussed in the General Discussion.

## Study 2

Study 1 did not support the third hypothesis that the effect of outcome salience on food choices is stronger for trials with short relative to long available response times. As pointed out, a potential limitation of the first study is that the number of participants and choice trials per participant was insufficient to reliably detect an interaction effect between the priming conditions and the available response time. To address this limitation, we conducted a second study similar to the first study but with three methodological changes. The first change was that the available response time was no longer manipulated continuously but binary, with two extreme categories of short and long available response times. Contrasting the extremes offers a clearer distinction between these two types of response times and increases the number of observations in each time condition. The second change is that we increased the number of choice trials. This was also done to increase the number of observations per participant. The third change is that Study 2 was conducted online. This allowed us to reach the target sample size during the COVID-19 pandemic, which was not possible in the laboratory-based study (Study 1). Furthermore, this resulted in a larger and demographically more diverse sample, thereby increasing the ecological validity of the findings.

The predictions were the same as in Study 1. We predicted that participants with higher health outcome values would make more healthy choices (Hypothesis 1a) and that those with higher taste outcome values would make more tasty choices (Hypothesis 1b). Further, we predicted more healthy choices in the health prime condition relative to the control prime condition (Hypothesis 2a) and more tasty choices in the taste prime condition relative to the control prime condition (Hypothesis 2b). Finally, we predicted that the difference in healthy choices between the health prime condition and the control prime condition would be larger for short than for long response times (Hypothesis 3a) and that the difference in tasty choices between the taste prime condition and the control prime condition would be larger for short than for long response times (Hypothesis 3b).

### Method

#### Transparency and openness

The method, materials, sample size estimate, inclusion criteria, hypotheses, and analysis plan of Study 2 were pre-registered on the Open Science Framework prior to data collection. This preregistration alongside all data and the analysis code are available at https://osf.io/kugbd (Koester et al. 2021). The computerized task was again programmed in PsychoPy (Peirce et al., 2019).

#### Participants

The target sample size of this study was 318 participants, which was determined with a pre-registered power analysis conducted with the MorePower 6.0.4 software (Campbell & Thompson, 2012). The analysis showed that 318 participants were required to reliably detect a small-to-medium effect size of *η*^*2*^*p* = 0.03, with statistical significance defined at the 0.05 level. Applying a stopping-rule during data collection, we recruited a total of 517 participants to reach the target sample size of 318 participants (106 per condition, 226 females, 89 males and 3 other, Age *M* = 33.1, *SD* = 10.8) that met the inclusion criteria (see below). The participants were UK residents collected through the participant pool of Prolific and were paid 2.10£ for taking part in the study. All participants provided informed consent, and the study was approved by the Social and Societal Ethics Committee at KU Leuven (G-2019 10 1766).

#### Inclusion criteria

As in the first study, we explicitly recruited participants who affirmed valuing a healthy diet while enjoying unhealthy food from time to time. Using the internal prescreening tool of Prolific, participants were only eligible for the study if they did not report any dietary restrictions. We only included participants if they did not eat within 2 h prior to the study. To ensure sufficient data points per participant, participants were only included in the analysis if they had at least 50 valid choice trials (the average number of valid trials per participant was 61.8). Just like in the first study, to qualify as a valid choice trial, the healthy item had to be rated as healthier and the tasty item as tastier at the end of the study. Finally, only trials on which participants responded before the response deadline were included.

#### Materials

We composed a set of 72 healthy and 72 tasty pictures, using the 60 healthy and 60 tasty items from Study 1 and 12 additional healthy and 12 tasty pictures. The latter pictures were added because the current study contained an additional block of choice trials.

#### Procedure

The second study was conducted online. At the start of the study, participants had to indicate how many hours ago they ate their last meal as well as their current hunger level on continuous scales from 0 to 10. Participants were then randomly and unknowingly assigned to a health, taste, or control condition.

**Fig. 3:**
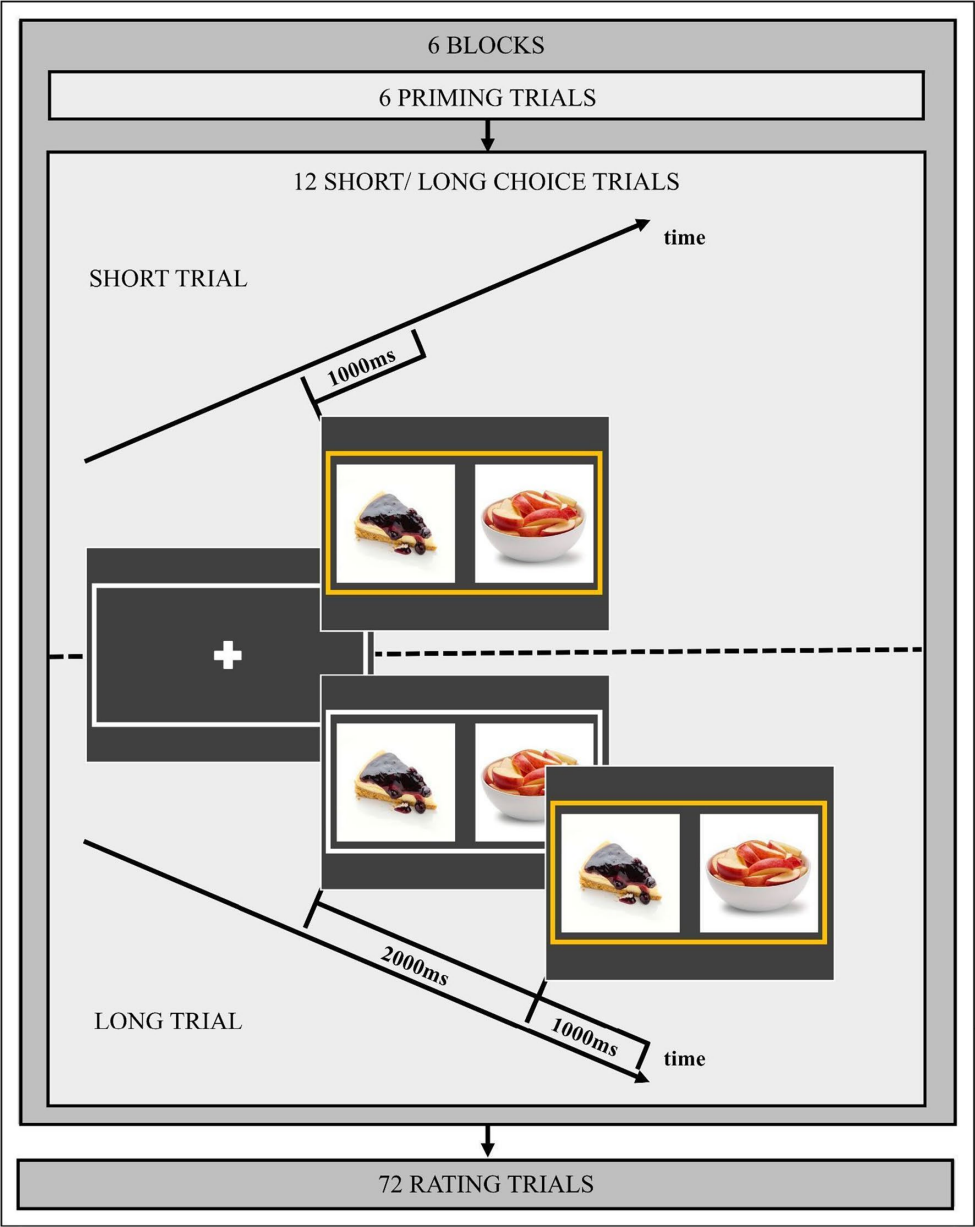
Experimental procedure of Study 2. *Note* The experiment had six blocks, each with six priming trials followed by 12 short or 12 long choice trials. These were followed by one block of 72 rating trials. The priming trials and rating trials were identical to those in Study 1 (see Fig. 1)

The rest of the procedure of the second study was similar to that of the first study and consisted of three parts. The first part was again made up of priming and choice trials, but to increase the number of data points, participants had to complete an additional block. This means that the first part consisted of six blocks, each made up of six priming trials followed by 12 choice trials (see Fig. 3). The priming trials were identical to the first study. The choice trials were similar to those in the first study except for the way in which the available response time was manipulated. Instead of manipulating it continuously, it was manipulated categorically with one short (1000 ms) and one long response time (3000 ms). Each block either consisted of 12 short or 12 long choice trials. At the start of each block, participants were informed whether the given block would consist of short or long trials. The order of blocks with short and long trials was counterbalanced across participants and conditions. The available response time was manipulated by instructing participants to respond as soon as the two food items were surrounded by a yellow frame and before this frame disappeared. On short trials, the frame and the food items appeared together from the start of the trial and disappeared after 1000 ms. On long trials, the frame appeared 2000 ms after the onset of the food items and disappeared again after 1000 ms. This means that participants had to respond within 1000 ms on short trials and within 2000–3000 ms on long trials. If participants responded after the items and frame had disappeared, they lost the opportunity to make a choice. In this case, participants were presented with a feedback message stating “Too slow”, and they nevertheless had to respond in order to proceed to the next trial. To familiarize participants with the procedure for the choice trials, they completed 12 short and 12 long practice trials with non-food items (i.e., flowers) prior to the first block of the actual study.

In the second part of the study, participants were asked to rate the healthiness and tastiness of each of the choice pairs presented in the first part. This part was identical to the second part of the first study with the exception that it consisted of 72 instead of 60 rating trials to accommodate the additional block of 12 choice trials that was added in the first part.

The third part of the study was identical to the third part of the first study. Participants were asked to rate their current hunger level, their health outcome values, and their taste outcome values, and to report any dietary restrictions.

### Results

#### Descriptive results

##### Health and taste outcome values

To check whether the values of health and/or taste outcomes differed across or between priming conditions, we conducted a mixed-model ANOVA to analyze the self-reported outcome values with priming condition (health, taste, control) as between-subjects factor and outcome value type (health vs. taste) as within-subjects factor.

The main effect of outcome value type was significant, *F*(1, 315) = 305.23, *p* < 0.001, *η*^*2*^_*p*_ = 0.49, indicating that participants valued a taste outcome (*M* = 8.82, *SD* = 1.50) more than a health outcome (*M* = 6.16, *SD* = 2.27). No other effects were significant, all *Fs* < 0.32, suggesting that health and taste outcome values did not differ between the priming conditions.

##### Hunger

To check whether there were differences in hunger between the priming conditions, we conducted a mixed-model ANOVA to analyze the self-reported hunger ratings with priming condition (health, taste, control) as between-subjects factor and time (pre-study vs. post-study) as within-subjects factor.

The main effect of time was significant, *F*(1, 315) = 130.21, *p* < 0.001, *η*^*2*^_*p*_ = 0.29, indicating that participants were less hungry at the start of the experiment (*M* = 4.88, SD = 2.66) than at the end (*M* = 5.86, SD = 2.96). No other effects were significant, all *Fs* < 1.50, suggesting no differences between conditions.

#### Food choices

To analyze the effects of health outcome value, taste outcome value, priming condition, and available response time on food choice, we conducted a mixed-model ANOVA on the proportion of healthy food choices with priming condition (health, taste, control) as between-subjects factor, available response time (short vs. long) as within-subjects variable, and centered health and taste outcome values as covariates.

In line with Hypothesis 1a, the main effect of health outcome value was significant, *F*(1, 312) = 22.21, *p* < 0.001, *η*^2^_*p*_ = 0.07, indicating that participants who reported higher values for the health outcome made more healthy choices. In line with Hypothesis 1b, the main effect of taste outcome value was significant, *F*(1, 312) = 4.53, *p* = 0.034, *η*^2^_*p*_ = 0.01, indicating that participants who reported higher values for the taste outcome made more tasty choices.

**Fig. 4:**
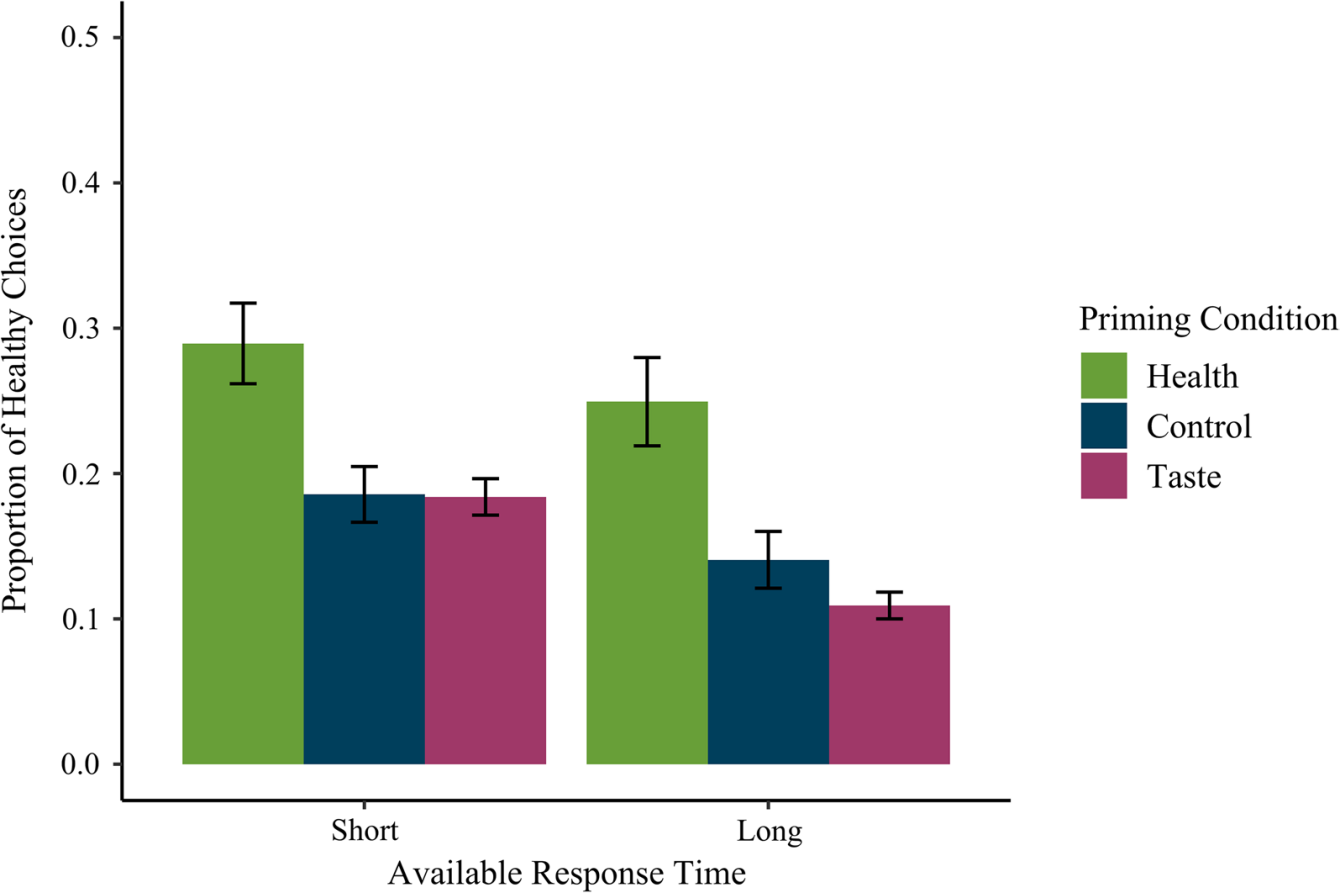
Proportion of healthy choices by available response time and priming condition. *Note* Error bars represent standard errors

In line with Hypothesis 2, the main effect of priming condition was also significant, *F*(2, 312) = 12.35, *p* < 0.001, *η*^2^_*p*_ = 0.07 (see Fig. 4). In line with Hypothesis 2b, post hoc comparisons indicated that participants in the health prime condition (*M* = 0.27, SD = 0.30) made more healthy choices than those in the control condition (*M* = 0.16, SD = 0.20), *t*(212) = 3.74, *p* < 0.001, and than those in the taste prime condition (*M* = 0.15, SD = 0.12), *t*(212) = 5.55, *p* < 0.001. Contrary to Hypothesis 2b, the difference between the control and taste prime conditions was not significant, *t*(212) = 1.04, *p* = 0.301.

Results also indicated a significant main effect of available response time, *F*(2, 312) = 57.49, *p* < 0.001, *η*^2^_*p*_ = 0.16, suggesting more healthy choices with short (*M* = 0.22, SD = 0.22) than with long available response times (*M* = 0.17, SD = 0.23; see Fig. 4).

Contrary to Hypothesis 3, the interaction between priming condition and available response time was not significant, *F*(2, 312) = 2.50, *p* = 0.084, *η*^2^_*p*_ = 0.02. No other effects were significant, all *Fs* < 3.46.

### Discussion

As in the first study, the findings of the second study support Hypothesis 1, that people make food choices in line with the values they attribute to health and taste outcomes. Participants who valued the health outcome more made more healthy choices, whereas those who valued the taste outcome more made more tasty choices. Furthermore, the second study replicated the finding of the first study that participants reported higher taste than health outcome values and made more tasty choices overall.

The findings of the second study partially support Hypothesis 2, that increasing the salience of a health/taste outcome via priming increases the likelihood that choices are made in line with this outcome. Consistent with this prediction and replicating the finding of the first study, priming participants with only health led to more healthy choices than priming them with both health and taste. However, contrary to the prediction and not in line with the findings of the first study, priming participants with only taste did not lead to more tasty choices than priming them with both health and taste.

As in the first study, the third hypothesis, that increasing the salience of a health/taste outcome via priming has a stronger effect on food choices with short relative to long decision times, was not supported. Instead, we replicated the finding of the first study that priming taste/health leads to an increase in tasty/healthy choices and that this effect is similar for short and long available response times. Thus, just like the first study, the second study was unable to provide support for the key assumption of the value accumulation account that increasing the salience of a health/taste outcome increases the likelihood that the outcome is considered first.

Interestingly, the second study also replicated the effect of the first study that all participants made more healthy choices with short relative to long response times, suggesting again that the health outcome was more likely to be considered on trials with short relative to long available response times.

## General discussion

Obesity continues to be on the rise and poses a number of serious negative consequences for individuals as well as for society at large. To address this problem effectively, it is important that individuals adopt a healthy diet. However, doing so can be challenging. Many individuals who explicitly state that health is important to them fail to make healthy food choices, especially under certain conditions such as when they are stressed or pressed for time. A promising explanation for unhealthy eating comes from the value accumulation account (see Berkman et al., 2017). This account puts forward a dynamic goal-directed process in which food choices are evaluated based on the sequential consideration of multiple outcomes of these choices, such as health and taste outcomes. This implies that food choices depend on the identity of the outcomes that are considered as well as on the order in which these outcomes are considered. It has further been proposed that both the identity and the order of the outcomes considered depend on the salience that these outcomes have. Thus, according to the value accumulation account, unhealthy food choices occur when only one outcome is considered—which is more likely when operating conditions are poor (e.g., time pressure)—and when the only outcome considered is the taste outcome.

We conducted two studies with the aim of testing three predictions derived from the value accumulation account. A first prediction is that food choices depend on the values that people ascribe to the outcomes of these choices. In line with this prediction, we found across both studies that participants reported higher values for a taste than a health outcome and made more tasty than healthy choices overall. Taken together, this suggests that people make more tasty choices because they value a taste outcome more than a health outcome. Another finding in line with this prediction was that individual differences in health and taste outcome values predicted food choices. Across both studies, participants who reported higher health outcome values made more healthy food choices. In the second but not the first study, participants who reported higher taste outcome values also made more tasty choices. As a potential reason for these different findings for health and taste outcome values, we noted that there was a lower variance in the self-reported taste than health outcome values. The low variance in taste outcome values may have reduced the explanatory power of taste outcome value as a predictor.

A second prediction derived from the value accumulation account is that increasing the salience of an outcome increases the likelihood that choices are made in line with this outcome. In support of this prediction, we found across both studies that health priming led to more healthy choices. In Study 1 but not in Study 2, we further found that taste priming led to more tasty choices. It should be noted, however, that in Study 1, participants reported higher hunger levels in the taste condition than in the other two conditions, making hunger a confounding variable for this finding. Taken together, these findings suggest that making health more salient has a stronger effect on food choices than making taste more salient, which is in line with previous findings (e.g., Hare et al., 2011). A possible explanation is that taste is likely to be chronically salient and thus at ceiling so that priming of this outcome has only a limited effect.

A third prediction derived from the value accumulation account is that increasing the salience of an outcome will increase the likelihood of choices in line with this outcome more strongly when time is short than when time is long. Contrary to this prediction, however, the priming effects that we obtained were independent of time across both studies. Priming health or taste affected choices similarly, whether participants were forced to make quick decisions or had more time. This suggests that increasing the salience of an outcome increases the likelihood that the outcome is considered regardless of whether there is time pressure or not. However, it does not support the assumption of the value accumulation account that increasing the salience of an outcome increases the likelihood that the outcome is considered first.

At this point, it may be worth revisiting the rationale for our Hypothesis 3, in particular, the idea that a stronger priming effect on food choices under short relative to long response times can be taken as evidence that the primed outcome was considered before the non-primed outcome. This interpretation rests on two assumptions. A first assumption is that time constraints increase the likelihood that the outcome that is considered first is also the only outcome that is considered before a decision is made. However, it is possible that time constraints were not stringent enough so that people were able to consider multiple outcomes under the imposed time constraints. Moreover, previous research has shown that people may increase accumulation speed when time is limited (e.g., Huseynov & Palma, 2021). A second assumption is that longer response times increase the likelihood that other outcomes besides the first outcome are considered as well. However, it is possible that longer response times did not increase the likelihood that other outcomes were considered. For instance, the likelihood to consider health may not increase with more time available after being primed with taste. In other words, a person who was primed with taste may never start to consider health, even when the person is given more time. If at least one of these assumptions does not hold, this can explain why similar priming effects were observed with and without time constraints.

As highlighted earlier, previous studies on time-dependent effects of priming have led to mixed results. Whereas Maier et al. (2020) found that the health priming effect was especially strong with short response times, this was not found in the study by Sullivan and Huettel (2021). Also in the current study we did not find evidence for this effect. Based on these mixed results, an examination of methodological differences seems warranted. An exhaustive comparison is beyond the scope of the current discussion, but we review three notable differences (see Table 2). A first difference is that all three studies used different priming procedures. Maier et al. (2020) relied on the data from a study in which participants were explicitly instructed to either consider the healthiness or tastiness of the food items while making decisions, or to make decisions naturally (Hare et al., 2011). Sullivan and Huettel (2021) asked participants to read about the importance of health and taste outcomes of food choices prior to the decision task. In our studies, we asked participants to rate food items prior to the decision task. This was done to make the priming procedure less obtrusive and thereby to reduce potential demand effects. Given that the health priming procedures were effective in all three studies, however, it seems unlikely that mixed effects across these studies can be explained by the specific priming procedures used.

**Table 2:** Methodological differences between studies examining the effect of priming on food choices across response times

	Priming procedure	Response time	Food preferences
Sullivan and Huettel (2021)	Read about importance of health or taste	Spontaneous	Binary
Maier et al. (2020)	Ask to consider healthiness, tastiness, or to decide naturally	Spontaneous	Single
Current studies	Rate items on healthiness, tastiness, or both	Forced	Binary

A second difference between the three studies has to do with the way in which the available response time was operationalized. We manipulated response times directly, whereas the other studies relied on differences in spontaneous response times. The fact that Maier et al. (2020) found a time-dependent priming effect indicates that the effect can be found with spontaneous response times. Yet, the fact that Sullivan and Huettel (2021) did not find the effect indicates that using spontaneous response times is not sufficient for finding the effect.

A third difference between the three studies concerns the way in which food preferences were assessed. In our studies and in that of Sullivan and Huettel (2021), food preferences were measured with binary choices. In the study of Maier et al. (2020), on the other hand, food preferences were measured with single items. It is thus possible that time-dependent priming effects are only observable when food preferences towards single items are measured but not when relative preferences between items are measured. To examine this possibility, future research could systematically manipulate the way in which food preferences are measured.

A notable finding in the two studies that we conducted is that participants made less healthy choices with more available response time across all three conditions. This deviates from the typical finding reported in previous studies that people make more healthy choices with more available response time (e.g., Sokol-Hessner, et al., 2012; Sullivan & Huettel, 2021; but see Schubert et al., 2021). As noted above, our studies differ from these previous studies in that we manipulated response times instead of relying on variations in spontaneous response times. Manipulating response times avoids confounds with other variables such as task difficulty, and arguably more closely resembles the real-life behavior of interest (i.e., making food choices when pressed for time). A potential implication of manipulating response times, however, is that time pressure may also inadvertently decrease accuracy in value-based choices (Milosavljevic et al., 2010). If this was the case in our studies, it could be part of the explanation for why we observed more healthy choices with less time. Across both studies, participants showed a preference for tasty items overall. If they were less accurate in expressing their preference under time pressure, this could explain why they made more healthy choices.

So far, we interpreted the priming effects that we obtained as the result of an increase in salience. It must be acknowledged, however, that the priming of an outcome may not only increase the salience of that outcome but also its value. At first blush, the finding that the values for taste and health outcomes measured at the end of our studies did not differ between priming conditions seems to speak against this idea. On closer thought, however, the fact that we did not have a baseline measure of taste and health outcome values prior to the priming procedure does not allow drawing definitive conclusions about this matter. Moreover, by asking participants about their health and taste values in food choices we may not have been able to capture momentary changes in values but chronic values instead**.** We therefore leave it open whether our priming effects were due to a temporary increase in salience, in value, or in both. It is important to note, however, that all three possibilities are still compatible with a value accumulation account in which choice behavior is based on a goal-directed weighing of the options and which is influenced by a temporary increase in salience and/or value.

Overall, our findings suggest that food choices are based on the values and the salience of health and taste outcomes even under poor conditions. They do not provide support for the sequential consideration of outcomes, and thus, further research is required. Yet while not all hypotheses by the value accumulation account were confirmed, our results are not better explained by traditional dual-process models of unhealthy food choices. In fact, several findings are incompatible with these traditional models. A first finding is that participants made less healthy choices with more available response time. According to traditional dual-process models, the opposite pattern would be expected. More time should allow goal-directed processes to operate, thus leading to more healthy choices. Another finding that seems at odds with traditional dual-process models is that priming of an outcome also led to more choices in line with this outcome when time was limited. This suggests that even under poor conditions, the outcomes of behavior options are considered and hence that the behavior is goal-directed rather than stimulus-driven.

The findings of this research hold important implications for our understanding of unhealthy eating as well as for the effectiveness of intervention strategies. Our findings suggest that people make value-based food choices. In particular, people tend to choose tasty items over healthy alternatives because they value a taste outcome more than a health outcome. One promising strategy to promote healthy eating from this perspective would be to offer alternatives that are both healthy and tasty. In this way, people may choose healthy items because these items satisfy the more important goal of eating something tasty. However, to achieve long-term behavior change and to promote healthy eating of moderately tasty items, it may also be important to increase the value people ascribe to health as an outcome of food choices. There seems to be growing awareness of the importance of a healthy diet already (Meyer, 2018). Carr and Epstein (2020) provide various ways in which the values of items can be manipulated to increase healthy food choices. Our current findings also suggest that health should be made salient in everyday settings in which food choices occur, such as in supermarkets and restaurants.

In the current research, we found support that making health temporarily more salient and/or valued may be a fruitful approach to increase healthy choices even when people are stressed or pressed for time. Other studies have found similar effects, suggesting that making healthy items perceptually salient can increase the proportion of healthy choices under poor operating conditions (Blom et al., 2021; Dai et al., 2020).

A limitation of the current research is that we did not measure individual differences with regard to weight and obesity (e.g., body mass index). Previous work has shown that high values for taste in individuals who are overweight or obese are associated with increased choice for willingness to work for tasty items (Giesen et al., 2010). This suggests that food choice measures, such as the ones in our studies, can provide a marker for real-world dieting behavior. However, given that our studies did not measure individual differences, they could not provide empirical support for this suggestion.

It may be worthwhile to consider the current research in its broader theoretical context. According to the traditional dual-process account, unhealthy choices are considered self-regulatory failures. It is assumed that they result from a stimulus-driven process in which no outcomes are considered. In the current research, we proposed that unhealthy food choices are not due to the absence of outcome processing but instead result from a dynamic goal-directed process that integrates multiple outcomes. We furthermore proposed that the consideration of outcomes depends on their salience and value, which in turn can be influenced through priming. The idea that unhealthy eating can be explained by considering multiple outcomes has also been emphasized by others (e.g., Fishbach et al., 2009; Kopetz & Orehek, 2015; Kruglanski et al., 2020). Similar to our proposal, it has been argued that unhealthy choices occur when certain outcomes (e.g., taste) gain dominance over other outcomes (e.g., health; Kruglanski et al., 2020). Based on such accounts, it has furthermore been questioned whether unhealthy choices should actually be considered a self-regulatory failure or rather a self-regulatory success with regard to this dominant outcome (Kopetz & Orehek, 2015). Similar to our proposal, others have also proposed that the influence of an outcome depends on the salience and importance of the outcome (Kruglanski et al., 2012) and that these can be influenced by contextual cues (Aarts & Dijksterhuis, 2000; Chartrand & Bargh, 1996; Ferguson & Bargh, 2004; Kruglanski et al., 2002; Moskowitz, 2002; Shah & Kruglanski, 2002).

Findings from the literature on Pavlovian-to-instrumental transfer can be regarded as empirical evidence for this idea. In these studies, it has been shown that the presentation of Pavlovian cues that are linked to a specific valued food outcome increase instrumental actions that are known to result in this outcome (Bray et al., 2008; Prevost et al., 2012). Thus, Pavlovian cues can be taken to prime certain valued outcomes. Importantly, PIT effects continue to depend on the value and availability of the primed outcome (for a discussion, see Mahlberg et al., 2019) and occur under poor operating conditions (e.g., under cognitive load; Seabrooke et al., 2018). Similar to our results, this suggests that choices in line with a primed outcome remain under goal-directed control even under poor operating conditions. Taken together, previous theoretical and empirical work seems to support a goal-directed explanation of unhealthy food choices.

Finally, it is important to note that the current research is unlikely to capture food choices in all their complexities. We wish to highlight three forms of complexity. First, the current study focused on health and taste outcomes of food choices. However, food choices are likely to be driven also by other outcomes such as their environmental impact, their social desirability, and their price. These outcomes may also hamper the choice of healthy food. Second, we focused on priming as a manipulation of outcome salience/values. However, outcome salience/values are likely to be influenced by a number of factors such as stress or negative affect. The salience/value of relevant outcomes such as taste may be temporarily increased because an individual is stressed or experiences negative affect and the consumption of something tasty is seen as an effective strategy to regulate stress or negative affect (Buabang et al., 2022; Mezuk et al., 2017; Polivy & Herman, 1993). Third, we focused on time as a factor constraining the consideration of outcomes in food choices. However, food choices may be constrained by other factors as well, such as cognitive load (Zimmerman & Shimoga, 2014). Individuals may be more likely to rely on the most salient/valued outcome and fail to consider other outcomes when they have many things on their mind. This being said, the general principles that motivated the current research can be applied to incorporate additional complexities of food choices and thereby point to other interesting avenues for future research.

## Conclusion

In order to effectively combat unhealthy eating, it is crucial to understand the underlying mechanisms of unhealthy food choices. According to the value accumulation account, behavior results from a dynamic goal-directed process in which behavior options are evaluated based on multiple outcomes that are considered sequentially over time. This account already received preliminary empirical support in previous research (Maier et al., 2020). The current work adds to this literature by showing that food choices are not only based on preexisting or chronic values of outcomes, but also depend on temporary increases in the salience and/or values of these outcomes. However, the effect of these temporary increases was not time-dependent. Thus, our results are inconclusive regarding the sequential consideration of outcomes. Instead, our findings showed that temporary increases in the salience and/or values of outcomes influenced choices with and without time pressure. This suggests nonetheless that such increases can already occur under time pressure. More research is needed to further investigate the sequentiality assumption of the value accumulation account.

## Supplementary Information

**Additional file 1:** Results for the empty model, constrained intermediate model, augmented intermediate model, and full model.

## Data Availability

The datasets generated and analyzed for all experiments reported in this manuscript are available at the Open Science Framework repository, https://osf.io/q8vh7 (Koester et al. 2021). All of the experiments were preregistered.

## Footnotes

1 The meaning of the term *outcome* corresponds to the term *attribute* that is sometimes used in the literature (e.g., Berkman et al., 2017).
