# Supplementary material for: A value accumulation account of unhealthy food choices: testing the influence of outcome salience under varying time constraints
Source: Cogn Res Princ Implic. 2023 Jan 12;8:4. doi: 10.1186/s41235-022-00459-6 (PMC9835743; doi:10.1186/s41235-022-00459-6)
Supplement: Supplementary file 1 — Additional file 1: Results for the empty model, constrained intermediate model, augmented intermediate model, and full model. [file 41235_2022_459_MOESM1_ESM.docx]

**Table 2**

*Results for the empty model, constrained intermediate model, and augmented intermediate model*

|  | Category | Empty Model | Constrained Intermediate Model (CIM) | | Augmented Intermediate Model (AIM) | |
| --- | --- | --- | --- | --- | --- | --- |
|  | | | *OR( 95% CI)* | *p* | *OR( 95% CI)* | *p* |
| Participant-level  Variables | | | | | | |
| Priming condition | Control |  | Reference | | Reference | |
|  | Health |  | 2.29 (1.52-3.45) | **< .001** | 2.13 (1.43-3.18) | **< .001** |
|  | Taste |  | 0.57 (0.37-0.86) | **.008** | 0.58 (0.39-0.88) | **.010** |
| Health Outcome Value |  |  | 1.27 (1.14-1.42) | **< .001** | 1.26 (1-13-1-40) | **< .001** |
| Taste Outcome Value |  |  | 0.89 (0.77-1.03) | .115 | 0.92 (0.80-1.06) | .253 |
| Hunger |  |  | 0.98 (0.89-1.07) | .632 | 0.96 (0.88-1.05) | .356 |
| Trial-level Variables | | | | | | |
| Available Response Time |  |  | 0.57 (0.49-0.67) | **< .001** | 0.52 (0.43-0.62) | **< .001** |
| Model Fit | | | | | | |
| ICCparticipant |  | 0.300 | 0.218 | | 0.161 | |
| AIC |  | 5667.42 | 5566.744 | | 5557.04 | |

*Note*. OR = odds ratio, CI = confidence interval, *p* = *p*-value

**Table 3**

*Results for the full model*

|  | Category | Full Model | |
| --- | --- | --- | --- |
| Participant-level  Variables | | *OR( 95% CI)* | *p* |
| Priming Condition | Control | Reference | |
|  | Health | 1.66 (0.98-2.82) | .060 |
|  | Taste | 0.61 (0.35-1.08) | .089 |
| Health Outcome Value |  | 1.25 (1.13-1.40) | **< .001** |
| Taste Outcome Value |  | 0.92 (0.80-1.06) | .258 |
| Hunger |  | 0.96 (0.88-1.05) | .359 |
| Trial-level Variables | | | |
| Available Response Time |  | 0.47 (0.23-0.51) | **< .001** |
| Cross-level  Interactions | | | |
| Priming Condition * Available Response Time | Control | Reference | |
| Priming Condition * Available Response Time | Health | 1.34 (0.89-2.02) | **.**162 |
| Priming Condition * Available Response Time | Taste | 0.95 (0.61-1.50) | **.**834 |
| ICCparticipant |  | 0.159 | |
| AIC |  | 5557.94 | |

*Note*. OR = odds ratio, CI = confidence interval, *p* = *p*-value
